# The relationship between sleep, gut microbiota, and metabolome in patients with depression and anxiety: A secondary analysis of the observational study

**DOI:** 10.1371/journal.pone.0296047

**Published:** 2023-12-20

**Authors:** Arisa Tanaka, Kenji Sanada, Katsuma Miyaho, Tomoyuki Tachibana, Shunya Kurokawa, Chiharu Ishii, Yoshihiro Noda, Shinichiro Nakajima, Shinji Fukuda, Masaru Mimura, Taishiro Kishimoto, Akira Iwanami

**Affiliations:** 1 Department of Psychiatry, Showa University Karasuyama Hospital, Tokyo, Japan; 2 Department of Neuropsychiatry, Keio University Hospital, Tokyo, Japan; 3 Institute for Advanced Biosciences, Keio University, Yamagata, Japan; 4 Intestinal Microbiota Project, Kanagawa Institute of Industrial Science and Technology, Kanagawa, Japan; 5 Transborder Medical Research Center, University of Tsukuba, Ibaraki, Japan; University of Rijeka Faculty of Medicine: Sveuciliste u Rijeci Medicinski fakultet, CROATIA

## Abstract

**Background:**

Growing attention is paid to the association between alterations in the gut microbiota and their metabolites in patients with psychiatric disorders. Our study aimed to determine how gut microbiota and metabolomes are related to the sleep quality among patients with depression and anxiety disorders by analyzing the datasets of our previous study.

**Methods:**

Samples were collected from 40 patients (depression: 32 patients [80.0%]); anxiety disorders: 8 patients [20.0%]) in this study. Gut microbiomes were analyzed using 16S rRNA gene sequencing and gut metabolomes were analyzed by a mass spectrometry approach. Based on the Pittsburgh Sleep Quality Index (PSQI), patients were categorized into two groups: the insomnia group (PSQI score ≥ 9, n = 20) and the non-insomnia group (PSQI score < 9, n = 20).

**Results:**

The insomnia group showed a lower alpha diversity in the Chao1 and Shannon indices than the non-insomnia group after the false discovery rate (FDR) correction. The relative abundance of genus *Bacteroides* showed a positive correlation with PSQI scores in the non-insomnia group. The concentrations of glucosamine and N-methylglutamate were significantly higher in the insomnia group than in the non-insomnia group.

**Conclusions:**

Our findings suggest that specific taxa could affect the sleep quality among patients with depression and anxiety disorders. Further studies are needed to elucidate the impact of sleep on specific gut microbiota and metabolomes in depression and anxiety disorders.

## Introduction

Dissatisfaction with sleep quality and quantity is highly prevalent in the general population [1, 2], and insomnia is considered a major public mental health problem [3]. Major depressive disorder (MDD) has complex relationships with insomnia, which is one of the diagnostic criteria according to the Diagnostic and Statistical Manual of Mental Disorders, Fifth Edition (DSM-5) [4]. Previous studies showed a high prevalence of sleep problems including insomnia, up to 90%, in patients with depression [5–7], and a recent cross-sectional survey based on the World Health Survey of 70 countries showed that sleep problems in patients with depression could lead to additional risks such as anxiety [8].

A growing attention has been paid to the field of gut microbiota including metabolomes, which represents the total low-molecular-weight chemical profile of metabolites in biofluids, cells, and tissues [9], in mental disorders over the last decade. We recently reported the characteristics of the gut microbiota in patients with MDD in a meta-analysis of 10 observational studies of MDD with a non-depressed group as controls [10]. Additionally, a recent meta-analysis reported diagnostic similarities of gut microbial disturbances in MDD, anxiety disorder, bipolar disorder, and schizophrenia [11]. On the other hand, a previous review reported an association between lack of sleep, the composition of gut microbiota, and gut microbial metabolomes in healthy adults as well as animals [12]. Especially with regard to MDD, a recent cross-sectional study reported several specific taxa related to sleep conditions in 36 patients with MDD compared to healthy controls [13]: the abundances of *Coprococcus* and *Intestinibacter* were associated with sleep quality independent of the severity of depression. Taken together, alterations in the composition of specific gut microbiota and their metabolomes may be associated not only with worsening depression but also with poor sleep quality.

However, to the best of our knowledge, very few studies have been conducted to explore the association between the composition of microbiota and metabolomes in the gut and sleep conditions for patients with psychiatric disorders. Zhang et al. [13] reported the association between sleep quality and the gut microbiota composition, not including the gut microbial metabolomes, in patients with MDD compared to healthy controls. Thus, we analyzed the data from our previous study [14] to examine the association between sleep and gut microbiota/metabolomes in patients with depression and anxiety disorders. Based on our hypothesis that there would be differences in bacterial and metabolomic features related to insomnia and non-insomnia in patients with depression and anxiety disorders, this study aimed to investigate the relationship between gut microbiota/metabolome characteristics and insomnia in patients with depression and anxiety disorders.

## Material and methods

### Ethics statement

This investigation was a secondary analysis of our multicenter prospective study that examined the effect of psychotropics on gut microbiomes in patients with depression and anxiety [14]. This trial was approved by the Institutional Review Board (IRB) of Showa University Karasuyama Hospital and Keio University School of Medicine and registered in the University hospital Medical Information Network (UMIN) Clinical Trials Registry (UMIN000021833). Before conducting the study, participants were fully informed about the procedures, and provided written consent. Detailed information was reported in our previously published paper [14].

### Subject recruitment and study procedure

From June 2017 to January 2018, participants were recruited at Showa University Karasuyama Hospital, Keio University Hospital, and Komagino Hospital in Tokyo. All participants including both males and females were 20 years of age or older with a diagnosis of depression and/or anxiety disorder based on the Diagnostic and Statistical Manual of Mental Disorders, Fifth edition (DSM-5). Exclusion criteria were as follows: (1) any organic gastrointestinal disorders; (2) receiving antibiotic medication; or (3) psychiatric symptoms that could be exacerbated by participation in the study. This is because functional gastrointestinal disorders such as irritable bowel syndrome have been reported to be associated with dysregulation of the gut microbiota-gut-brain axis [15], but organic gastrointestinal disorders are thought to be more affected by the disease itself than by the gut microbiota, which may have a greater impact on sleep. Furthermore, antibiotics have the most direct and significant impact on the gut microbiota, which may also affect the sleep condition.

For inpatients, fecal samples were collected, and psychiatric assessments were conducted at three time points during hospitalization. Baseline assessments were conducted within 10 days of admission; midterm assessments were conducted between 14 and 20 days after admission; and endpoint assessments were conducted between 21 days after admission and discharge. Each assessment point required a period of at least one week. For outpatients, a maximum of three fresh stool samples were collected, and psychiatric assessments were conducted on three consecutive visits. For both inpatients and outpatients, fecal samples were collected a maximum of three times at each time point to reduce the fluctuating impacts of the patient’s daily dietary and sampling bias. In all cases, if more than one sample was collected, the mean value of the data was used. Trained psychiatrists and psychologists evaluated the severity of psychiatric symptoms using the Pittsburgh Sleep Quality Index (PSQI) [16] at baseline and endpoint, the Hamilton Depression Rating Scale (17 items) (HDRS) [17], and the Hamilton Anxiety Rating Scale (HAM-A) [18] at each time point. The PSQI is a self-report questionnaire used to assess subjective sleep quality and consists of seven components (each component ranges from 0 to 3): sleep quality, sleep latency, sleep duration, sleep efficiency, sleep disturbance, sedative-hypnotic drugs, and daily life functioning [16].

Based on the PSQI scores, participants were classified into two groups: insomnia and non-insomnia groups. The insomnia group was defined as having a PSQI score ≥ 9 at baseline, while the non-insomnia group was defined as having a PSQI score < 9 at baseline [19]. In this study, only data from the baseline assessment was used. The longitudinal data from the midterm and endpoint assessments was presented in our previous papers [14, 20, 21].

### Fecal sample collections and DNA extraction

For both inpatients and outpatients, fresh fecal samples were frozen immediately on collection and transported to the laboratory within 48 hours, especially outpatients were transported by courier service. The samples were stored in a freezer at -80°C for further analyses. The 16S rRNA gene was analyzed by the following method [22]. First, fecal samples were lyophilized for 12–18 hours using a VD-800R lyophilizer (TAITEC, Nagoya, Aichi, Japan). Then, each freeze-dried fecal sample was combined with four 3.0 mm zirconia beads and subjected to vigorous shaking (1500 rpm for 10 min) using a Shake Master (Biomedical Science, Shinjuku, Tokyo, Japan). Second, approximately 10mg of each fecal sample was combined with approximately 100 mg of 0.1-mm zirconia/silica beads, 300μL DNA extraction buffer (TE containing 1% (w/v) sodium dodecyl sulfate), and 300μL of phenol/chloroform/isoamyl alcohol (25:24:1), and subjected to vigorous shaking (1500 rpm for 5 min) using a Shake Master. The resulting emulsion was subjected to centrifugation at 17,800×g for 10 min at room temperature, and bacterial genomic DNA was purified from the aqueous phase by a standard phenol/chloroform/isoamyl alcohol protocol. RNA was extracted from the sample by RNase A treatment. The resulting DNA sample was then purified again by another round of phenol/chloroform/isoamyl alcohol treatment and ethanol precipitation by GENE PREP STAR PI-480 (Kurabo Industries Ltd., Osaka, Japan). V1-V2 hypervariable region of 16S rRNA encoding genes were amplified by Polymerase Chain Reaction (PCR) using a bacterial universal primer set [23, 24]. The amplicons were analyzed using a Miseq sequencer (Illumina, San Diego, California, U.S.A.) with some modifications previously indicated [22]. The filter-passed reads were then processed using the Quantitative Insights into Microbial Ecology (QIIME) 2 (2019.10) [25]. Denoising and trimming of sequences were processed using DADA2. 20 bp and 19 bp were trimmed from 5’ ends of forward and reverse reads respectively to remove primer sequence. 280 bp and 210 bp length reads from 5’ ends were used for further steps. Sequences were clustered into operational taxonomic units (OTUs) that reached 97% nucleotide similarity, and OTUs were assigned to the SILVA 132 database [26, 27] using the Naive Bayesian Classifier algorithm. Alpha diversity of the gut microbiota was calculated using three parameters: Chao1, Shannon, and the whole tree index of Phylogenetic diversity (PD). Principal coordinate analysis (PCoA) based on weighted and unweighted UniFrac distance and analysis of similarity (ANOSIM) tests was conducted using the QIIME 2. Linear discriminant analysis (LDA) effect size (LEfSe) was used to analyze the characteristics of different abundances [28]. Cladograms were drawn using the Huttenhower Galaxy web application (The Huttenhower Lab, Boston, USA) [28].

### Metabolomic analysis

For metabolomic analysis using capillary electrophoresis time-of-flight mass spectrometry (CE-TOFMS), the Human Metabolome Technologies (HMTs; Tsuruoka, Yamagata, Japan) standard library [29] were used to analyze cationic and anionic metabolites in positive and negative modes. In short, fecal metabolites were extracted from 10 mg of frozen feces by vigorous shaking with internal standards. To this mixture, 100 mg of 0.1 mm zirconia/silica beads (BioSpec Products, Bartlesville, OK, USA) were bottled and vigorously stirred for 5 min using a Shake Master NEO (Biomedical Science, Shinjuku, Tokyo, Japan). The mixture was then dripped with solution (500 μL of chloroform and 200 μL water extraction), the suspension was centrifuged at 4,600×g for 15 minutes at 4°C, and the resulting supernatant was transferred to a 5-kDa-cutoff filter column (Ultrafree MC-PLHCC 250/pk, HMT). After drying the flow-through under vacuum, the residue was dissolved in 50 μL of Milli-Q water containing the reference compounds (200 μM each of 3-aminopyrrolidine and trimesate). All CE-TOFMS experiments were performed using an Agilent capillary electrophoresis system (Agilent Technologies, Santa Clara, CA, USA). Raw data were analyzed using our proprietary automatic integration software (MasterHands ver. 2.16.0.15 developed at Keio University) [30]. Annotation tables were created from the measurements of the standard compounds and were aligned with the datasets based on similar m/z values and normalized migration time (MT). The areas of the annotated peaks were then normalized based on internal standard levels and sample volume to obtain the relative levels of each metabolite.

### Statistical analysis

We used statistical software IBM SPSS Statistics version 25.0 (SPSS Inc. Chicago, IL, USA). Categorical variables were described as the mean ± standard deviation (S.D.) and number (%), respectively, and the distribution of the data was checked using histograms, q-q plots, and Shapiro-Wilk tests before conducting statistical analyses. For categorical variables (sex and diagnosis), Pearson Chi-squared test was used to compare between the insomnia and non-insomnia groups. For all statistical tests, the significance level was set to 0.05. Independent t-tests were used to compare differences in demographics and participants characteristics. Multiple linear regression analysis was performed to analyze alpha diversity (Chao1, Shannon, and PD whole tree indices) and metabolites between the insomnia and non-insomnia groups with diagnosis (depression or anxiety) and group (insomnia or non-insomnia) as dependent variables and with alpha diversity index and metabolites as independent variables. LDA scores were calculated for taxa that were differentially abundant at the family and genus levels between the insomnia and non-insomnia groups. LEfSe analysis was performed by Monte-Carlo method (*alpha* = 0.05) between-class factorial Kruskal-Wallis test, and the threshold for the logarithmic LDA score of discriminative features was 4.0. LEfSe analysis was used to identify microbial taxa that differed in abundance between the insomnia and non-insomnia groups. In cases where there was a significant difference in microbial taxa or metabolites between the insomnia and non-insomnia groups, Mann-Whitney U-test was performed to assess the difference in each microbial taxon or each metabolite, and statistical corrections were conducted by controlling the false discovery rate (FDR) through the Benjamini-Hochberg procedure [31] with alpha set at 0.05. As exploratory analyses, Spearman’s correlation coefficients were conducted between the microbial taxa with significant differences and PSQI scores.

## Results

**Fig 1:**
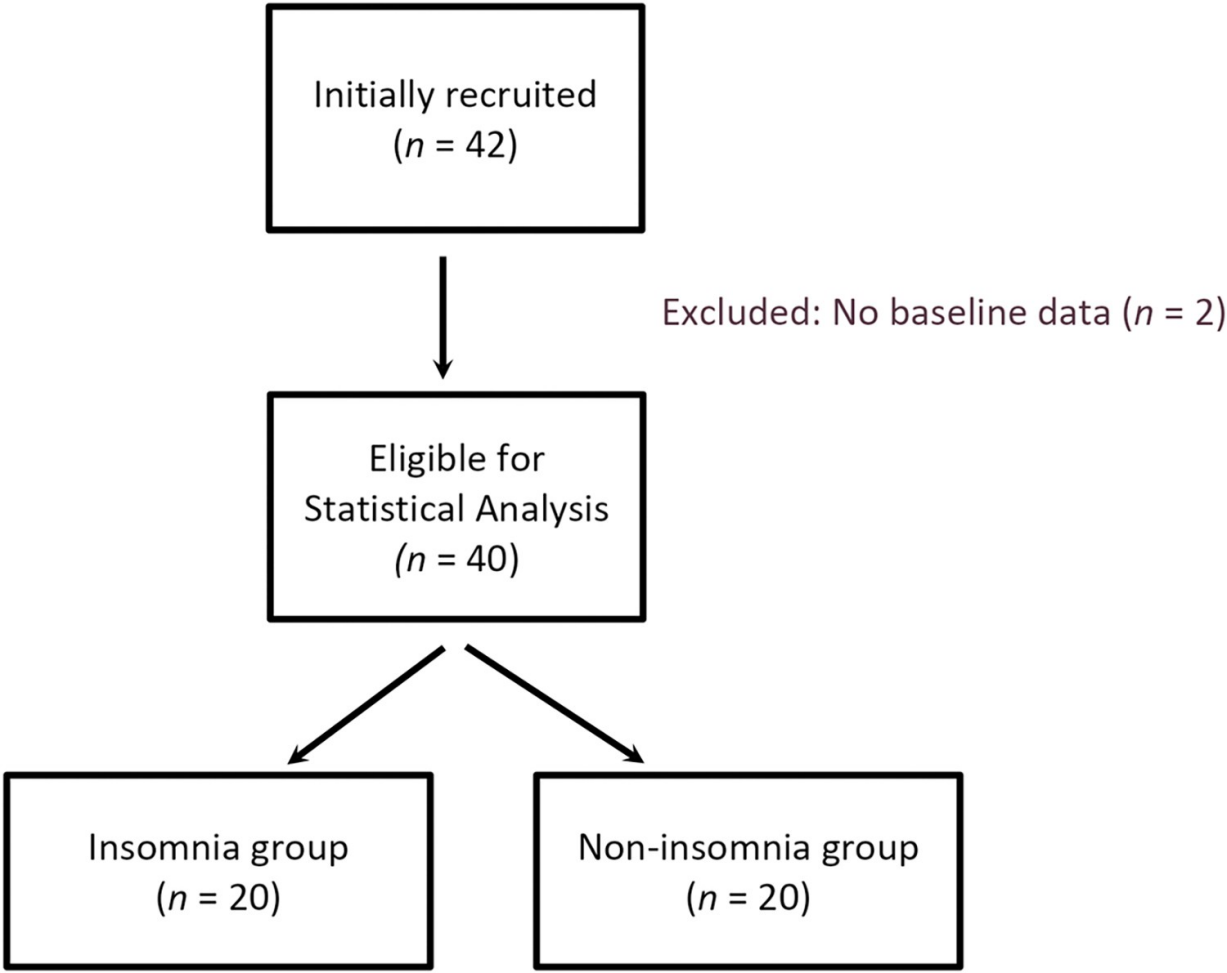
Flow chart of participants through the trial.

The flowchart of the study is shown in Fig 1. Of the 42 participants initially recruited as 14 inpatients (33.3%) and 28 outpatients (66.7%), two participants did not undergo baseline assessments. Thus, 246 fecal samples were collected from the 40 participants (23 females [57.5%] and 13 inpatients [32.5%]) who completed the baseline assessments.

### Clinical characteristics

Clinical characteristics of the participants included in the study are shown in Table 1. Of the included 40 participants, twenty-four patients (60.0%) were diagnosed with MDD, 8 (20.0%) with persistent depressive disorder, 6 (15.0%) with general anxiety disorder, 1 (3.0%) with panic disorder, and 1 (3.0%) with social anxiety disorder. In addition, 12 patients (30.0%) were diagnosed with both MDD and anxiety disorders. When the participants were classified based on PSQI scores, 20 participants (50.0%, 7 males and 13 females) were in the insomnia group and 20 participants (50.0%, 10 males and 10 females) were in the non-insomnia group. There were no significant differences in age, BMI, HAM-A, or HDRS between the two groups. The PSQI score and the percentage of depression were higher in the insomnia group than in the non-insomnia group (*p* < 0.001, *p* < 0.001, respectively).

**Table 1:** Baseline characteristics of patients stratified by insomnia.

	Insomnia (*n* = 20)	Non-insomnia (*n* = 20)	*χ2*	*p* value
	*n*	*n*		
**Male (%)**	7 (35.0%)	10 (50.0%)	0.672	.180
**Feale (%)**	13 (65.0%)	10 (50.0%)	0.391	.532
**Depression (%)**	18 (90.0%)	14 (70.0%)	0.985	**< .001**
	***mean (SD)***	***mean (SD)***	***t***	***p* value**
**Age (year)**	56.7 (17.1)	53.9 (21.5)	0.467	.643
**BMI (Kg/m** ^**2**^ **)**	22.7 (3.8)	21.6 (4.7)	0.879	.385
**PSQI**	13.3 (3.5)	6.3 (1.8)	8.193	**< .001**
**HDRS**	19.1 (6.8)	10.2 (6.2)	1.460	.153
**HAMA**	18.5 (6.7)	10.4 (7.4)	0.871	.389

Significant differences are presented in bold.

Abbreviations: BMI, Body Mass Index; HAMA, Hamilton Rating Scale for Anxiety; HDRS, Hamilton Depression Rating Scale; PSQI, the Pittsburgh Sleep Quality Index; SD, standard deviation.

### Comparison of alpha diversity between the groups

**Fig 2:**
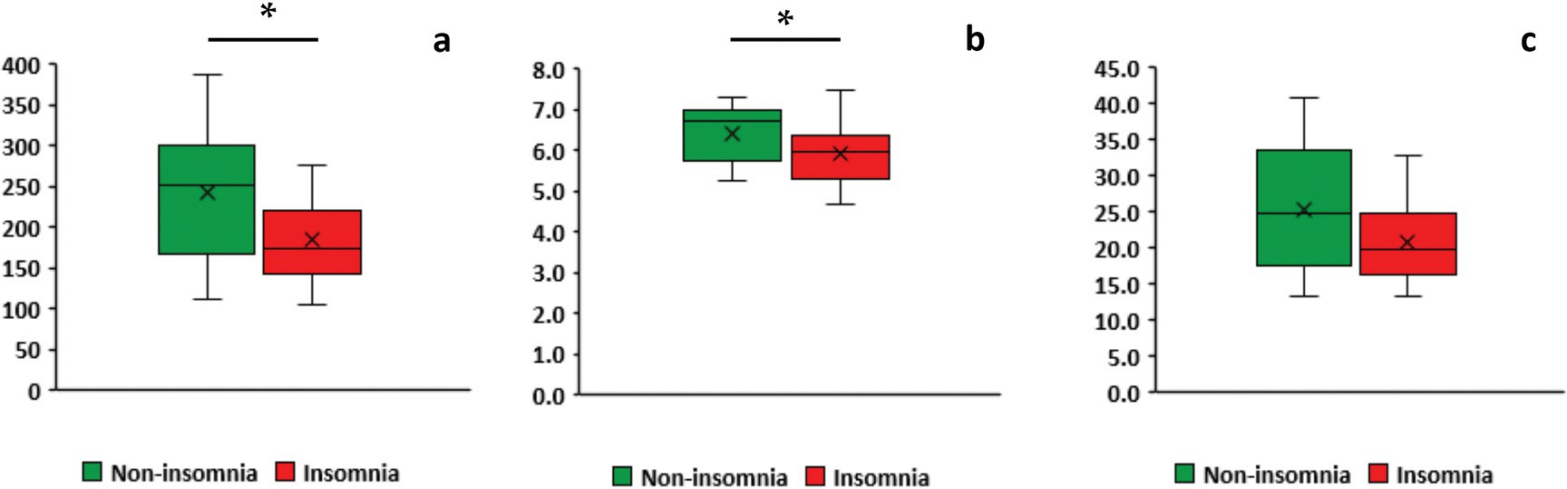
Microbiota alpha diversity between insomnia and non-insomnia groups using the box plot. (a) Chao 1 index. (b) Shannon index. (c) PD whole tree. The box shows the upper quartile and lower quartile. The median is indicated by a line. The whiskers extend from maximum to minimum data. Asterisks (*) indicate statistically significant.

As shown in Fig 2, the insomnia group showed a lower diversity in the Chao1 and Shannon indices compared with the non-insomnia group (Chao1: *p* = 0.031, Shannon: *p* = 0.036, respectively), which remained significant after the FDR correction (*p* = 0.036, *p* = 0.036, respectively).

### Comparison of beta diversity between the groups

Beta diversity of the samples in each group was shown in S1 Fig. ANOSIM with permutations confirmed no significant separation of groups in weighted and unweighted UniFrac distances, indicating that there were no clear differences in the structure of the bacterial community between each group.

### Comparison of bacterial composition between the groups

**Fig 3:**
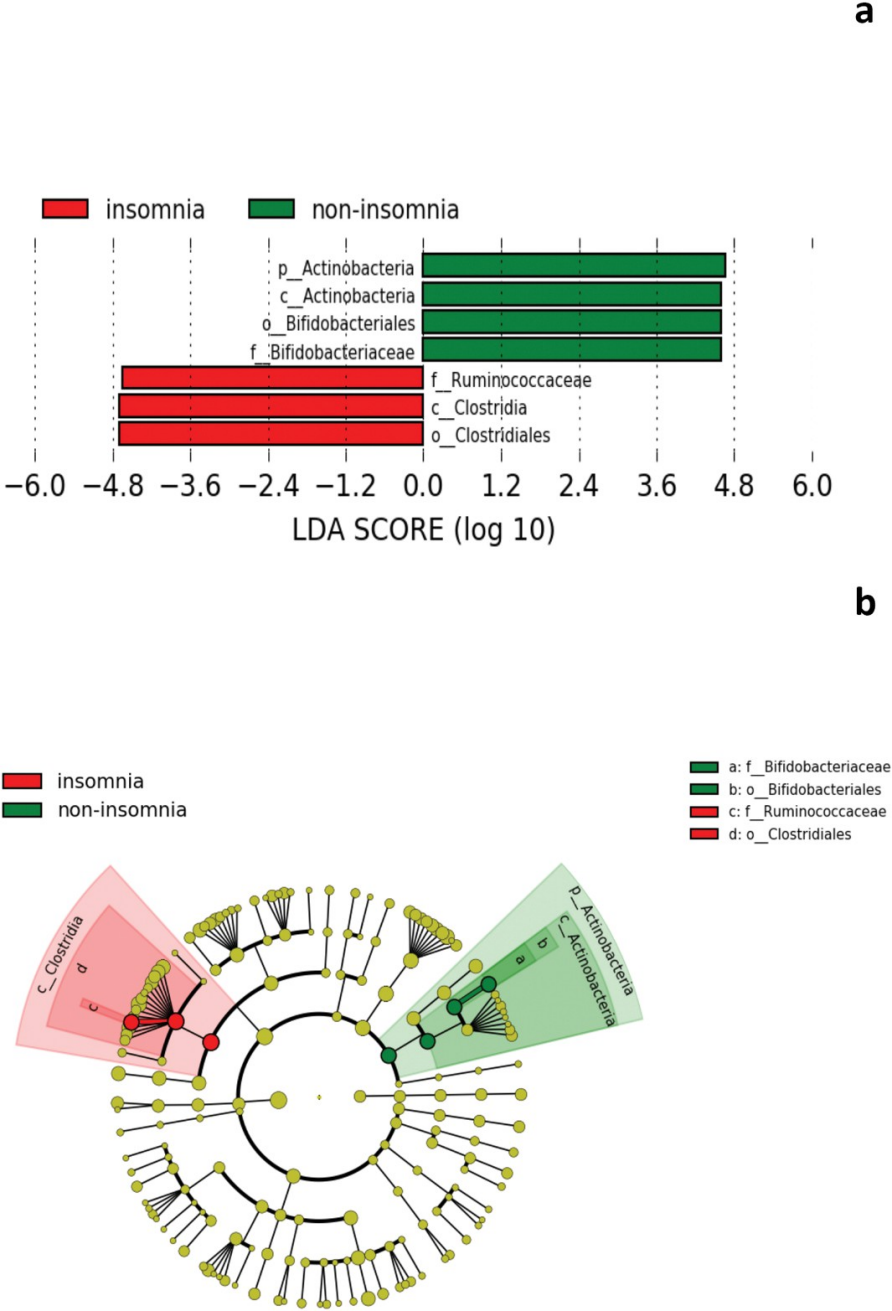
Specific bacterial taxa at family level between insomnia (red) and non-insomnia (green) groups. (a) LDA score. (b) Cladogram. Abbreviations: f = family level, g = genus level, o = order level.

At the family level, Ruminococcaceae showed a trend-toward higher prevalence in the insomnia group compared to the non-insomnia group, and conversely, Bifidobacteriaceae showed a trend-toward higher prevalence in the non-insomnia group compared to the insomnia group (Fig 3). However, these differences did not reach statistical significance after the FDR correction.

**Fig 4:**
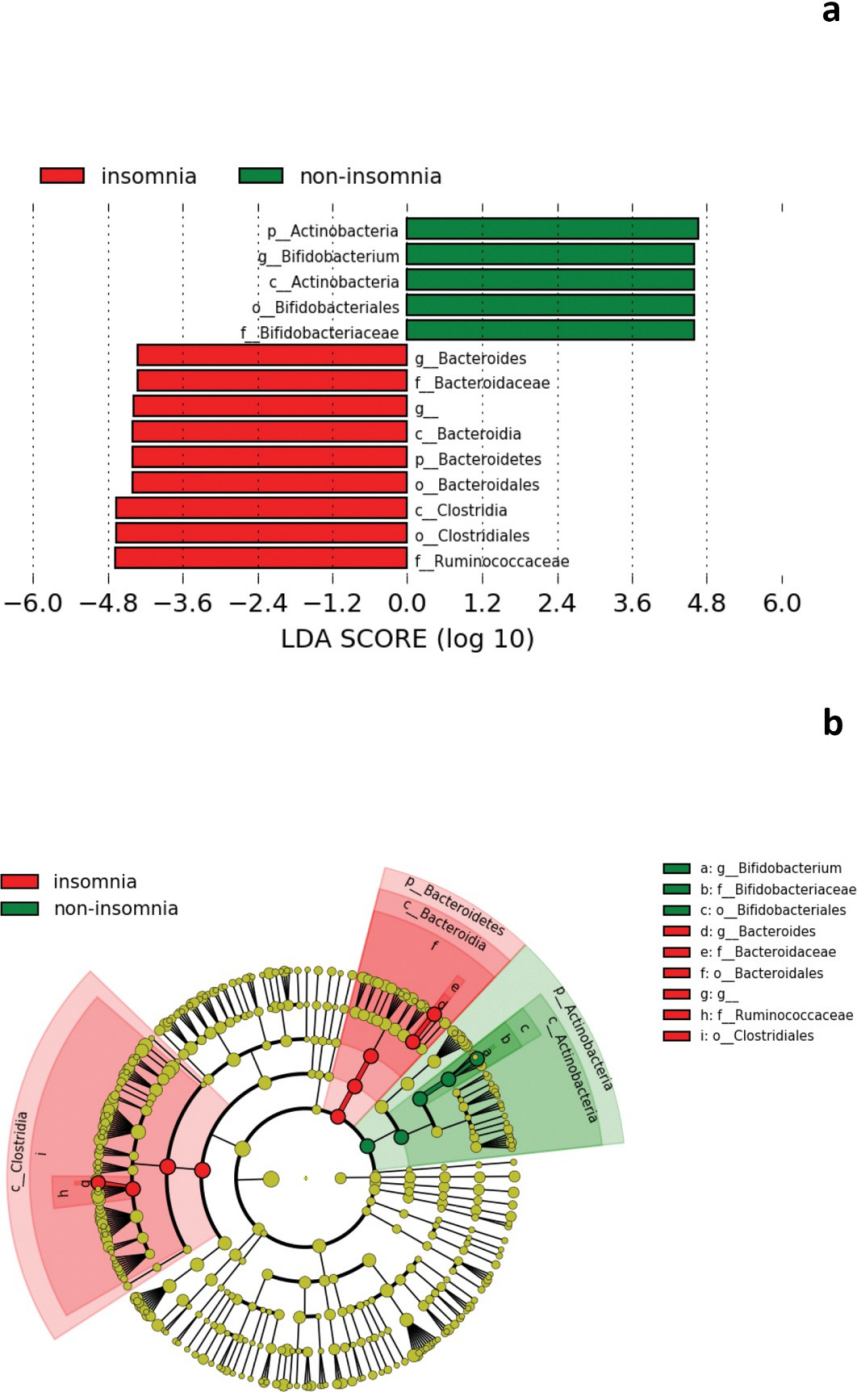
Specific bacterial taxa at genus level between insomnia (red) and non-insomnia (green) groups. (a) LDA score. (b) Cladogram. Abbreviations: f = family level, g = genus level, p = phylum level.

**Fig 5:**
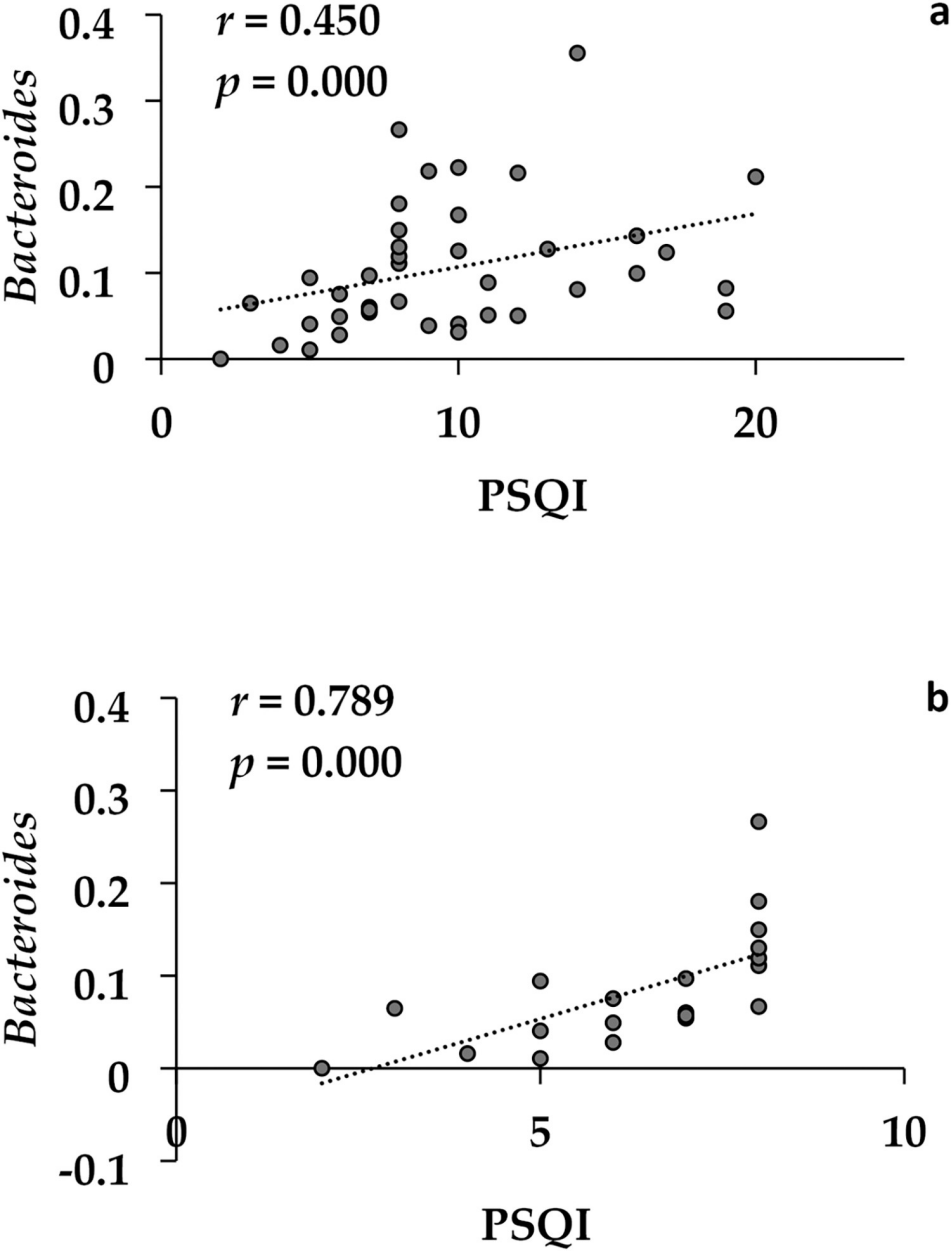
A correlation between PSQI scores and the relative abundance of genus *Bacteroides* among all patients (n = 40) (a) and non-insomnia group (n = 20) (b).

At the genus level, *Bacteroides* showed a trend-toward higher relative abundance in the insomnia group compared to the non-insomnia group, and conversely, *Bifidobacterium* showed a trend-toward higher prevalence in the non-insomnia group compared to the insomnia group (Fig 4). However, these differences did not reach statistical significance after the FDR correction. On the other hand, *Bacteroides* showed a correlation with PSQI scores (*r* = 0.450, *p* < 0.001) at baseline among all patients, and it showed a correlation with PSQI scores (*r* = 0.789, *p* < 0.001) particularly in the non-insomnia group (Fig 5).

### Comparison of metabolomic composition between the groups

**Fig 6:**
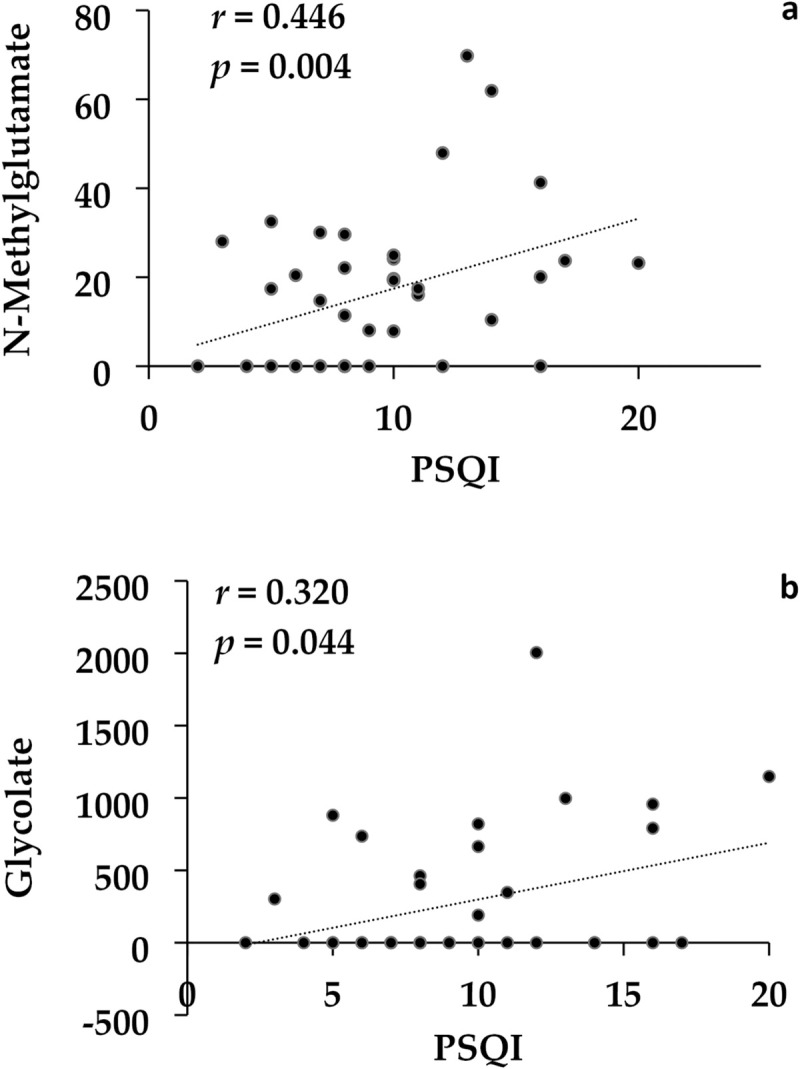
A correlation between PSQI scores and concentrations of N-methylglutamate (a) and glycolate (b) among all patients (n = 40).

Total 514 metabolites were analyzed for the 40 participants. The multiple regression analysis demonstrated significantly different metabolite concentrations between the insomnia and non-insomnia groups (Table 2). The concentrations of glucosamine and N-methylglutamate were higher in the insomnia group than in the non-insomnia group, which remained significant after the FDR correction. The concentration of glycolate was higher in the insomnia group than in the non-insomnia group, which did not survive after the FDR correction. Concentrations of N-methylglutamate and glycolate showed significant correlations with PSQI scores among all the patients (*r* = 0.446, *p* = 0.004; *r* = 0.320, *p* = 0.044, respectively), which remained significant after the FDR corrections (*p* = 0.008; *p* = 0.044, respectively) (Fig 6).

**Table 2:** Significantly different metabolites between insomnia and non-insomnia groups.

	Insomnia (*n* = 20)	Non-insomnia (*n* = 20)	Uncorrected *p* value	FDR corrected *p* value
**Glucosamine**	87.7 (67.5)	44.1 (22.0)	**.012**	**.036**
**Glycolate**	422.7 (555.2)	139.3 (271.3)	**.036**	n.s.
**N-Methylglutamate**	24.6 (20.6)	10.3 (12.7)	**.012**	**.036**

Values are presented as mean and standard deviation.

Abbreviations: FDR, false discovery rate; n.s., not significant.

## Discussion

This is an exploratory study to evaluate the association between sleep conditions, gut microbiota/metabolites composition in patients with depression and anxiety disorders. Our findings revealed that the insomnia group had lower alpha diversity in the Chao1 and Shannon indices compared to the non-insomnia group. A higher relative abundance of genus *Bacteroides* was correlated with higher PSQI scores in the non-insomnia group. Additionally, the concentrations of glucosamine and N-methylglutamate were higher in the insomnia group than in the non-insomnia group.

We found differences in baseline gut bacterial diversity between the insomnia and non-insomnia groups, with the insomnia group exhibiting significantly lower diversity according to the Chao 1 and Shannon indices. Previous studies have also reported a significant decrease in alpha diversity in the Chao1 [32, 33] and Shannon [34] indices in insomnia patients compared to healthy controls (HCs), consistent with our findings. In addition, studies have demonstrated a significant decrease in OTU richness under sleep restriction in rats compared with their baseline [35], and in observed amplicon sequence variants under sleep restriction in healthy men compared with adequate sleep time [36]. A plausible mechanism of this relationship between the bacterial diversity and sleep is that a decrease in melatonin due to the lack of sleep causes a decreased anti-inflammatory cytokines and increased pro-inflammatory cytokines, which in turn injure the colonic mucosal, leading to a decreased diversity and richness of the gut microbiota in mice [37]. On the other hand, the PCoA plots based on weighted and unweighted UniFrac distances showed no significant differences in the structure of bacterial communities at baseline between the insomnia and non-insomnia groups. Similar to our findings, previous studies reported that beta diversity was not altered by sleep restriction in healthy subjects [35, 38]. In contrast, a recent study showed significant differences in the structure of bacterial communities among African-origin adults in response to sleep length [39]. Thus, future studies should further explore the relationship between gut bacterial alpha and beta diversity indices and sleep duration in patients with psychiatric disorders.

It has been considered that sleep and gut microbiota are bidirectionally linked through inflammation [12]; the lack of sleep stimulates the hypothalamic-pituitary-adrenal axis, which results in a release of corticosterone [40], that increases inflammation mediated through the gut microbiota [41]. Our study found that the relative abundance of family Ruminococcaceae at baseline was significantly higher in the insomnia group, whereas family Bifidobacteriaceae was significantly higher in the non-insomnia group at baseline. Ruminococcaceae is a core bacteria family that exists in the human intestine, and its relative abundance tends to decrease with aging [42]. Zhang et al. [43] reported an increase in the abundance of Ruminococcaceae in hypertensive patients with sleep apnea compared to those without sleep apnea. In contrast, Jiang et al. [44] reported lower levels of *Ruminococcaceae UCG-002* and *Ruminococcaceae UCG-003* in the chronic insomnia group compared to the healthy group, which is inconsistent with our finding. A recent study also reported that the relative abundance of family Ruminococcaceae was significantly decreased under sleep restriction in healthy men compared with adequate sleep time [36]. Regarding family Bifidobacteriaceae, it is the only family member of the order Bifidobacteriales within the phylum Actinobacteria and contains the genus *Bifidobacterium* [45]. In support of our findings, some *Bifidobacterium* species could produce gamma-aminobutyric acid (GABA) [46], which could promote sleep by activating GABA_A_ receptors [47]; moreover, a preclinical study have shown a decreased relative abundance of *Bifidobacterium* in sleep-disrupted mice compared to control mice [48]. Taken together with prior studies and our results, Bifidobacteriaceae may be associated with sleep conditions. We found that the relative abundance of the genus *Bacteroides* was significantly higher in the insomnia group, and a higher relative abundance of *Bacteroides* was significantly correlated with higher PSQI scores at baseline among all patients. *Bacteroides* is a genus of bacteria that is a major constituent of the gut microbiota [49]. In support of our finding, Li et al. [32] reported an increased relative abundance of *Bacteroides* in insomnia patients compared with HCs; in contrast, another previous studies showed a lower proportion of *Bacteroides* in the insomnia group compared with HCs [34, 50]. These discrepancies could be due to differences in the methods of genetic analysis (e.g., 16S rRNA sequencing, region, qPCR) and age of target subjects (i.e., young, middle-aged) [21]. Given that the correlation between sleep conditions and the gut microbiota remains controversial, further studies focusing on the relationship between the gut microbiota and sleep in patients with psychiatric disorders are needed.

It has been proposed that gut metabolites, specifically short-chain fatty acids, may influence clock gene expression; gut microbiota as well as its metabolites directly modulate the circadian rhythm [51]. In this study, the insomnia group had higher levels of glucosamine and N-methylglutamate, and glycolate and N-methylglutamate correlated significantly with PSQI scores among all patients. Glucosamine is a popular dietary supplement [52] found in various forms, including N-acetylglucosamine (GlcNAc) [53, 54], but its relationship with sleep conditions in clinical settings has not been studied before while previous preclinical studies reported that O-linked b-N-acetylglucosamine (O-GlcNAc) was a new metabolomic signal linking metabolism to the circadian clock [55–57]. On the other hand, glutamate, the most abundant excitatory neurotransmitter in the brain, plays an important role in the regulation of sleep and wakefulness, that is, sleep homeostasis [58, 59]. Previous studies have reported the association between glutamate levels and sleep conditions in various psychiatric disorders, including MDD [60], schizophrenia [61], autism spectrum disorder [62], alcohol use disorder [63], and posttraumatic stress disorder (PTSD) [64], but it remains unclear whether the glutamate levels are due to the psychiatric disorder or coexisting sleep disorders. Additionally, a recent study in mice showed that the lack of vitamin B2 (VB2) increased glycolate (glycolic acid) concentrations in the gut microbiota and the host [65], and a human study found that lower VB2 intake was associated with low sleep quality [66]. While our results suggest a possible association between fecal metabolomes and sleep conditions in patients with depression and anxiety disorders, further studies are warranted to identify specific fecal metabolomes related to sleep conditions in psychiatric disorders.

The results of this study have several limitations. First, the sample size of this study was small. To evaluate the more complex associations between gut microbiomes/metabolomes and sleep, and to avoid beta errors, a large cohort study would be necessary [14, 20, 21]. Second, we did not consider the impact of environmental factors on the composition of gut microbiota in each patient. The content of diet, smoking, drinking alcohol, as well as exercising habits affect the gut microbiota [67–71]. In the present study, the environmental factors may have influenced our findings. Third, we did not consider the effects of prescribed medicine taken before participating in the current study. Our previous study has suggested that antipsychotics may decrease alpha diversity in the gut microbiota among patients with depression and anxiety [14]. Additionally, a recent research has shown that the gut microbiota may influence the metabolism of hypnotic drugs [72]. Finally, the patient’s past physical problems were not considered, despite the fact that endocrine diseases (e.g., diabetes, thyroid hormone abnormalities) are known to be associated with alterations in the gut microbiota [73–75].

Despite a number of limitations, we demonstrated that the insomnia group showed a lower alpha diversity in the Chao1 and Shannon indices compared with the non-insomnia group, similar to previous studies. Additionally, although not consistent with previous studies, *Bacteroides* showed a positive correlation with sleep quality in the non-insomnia group. We further have demonstrated for the first time to our knowledge that the levels of glucosamine and N-methylglutamate were significantly higher in the insomnia group than in the non-insomnia group. Given an exploratory nature of this study, there is need to further investigate the potential relationship between specific taxa, metabolomes, and sleep disturbance in psychiatric disorders.

## Supporting information

S1 Fig Microbiota beta diversity of samples in the insomnia group (blue) and non-insomnia group (orange) by PCoA (Principal coordinate analysis) based on unweighted (a) and weighted (b) UniFrac distances and analysis of similarity (ANOSIM) tests.
(TIF)
