# Supplementary figures and images for: The relationship between sleep, gut microbiota, and metabolome in patients with depression and anxiety: A secondary analysis of the observational study
Source: PLoS One. 2023 Dec 20;18(12):e0296047. doi: 10.1371/journal.pone.0296047 (PMC10732403; doi:10.1371/journal.pone.0296047)

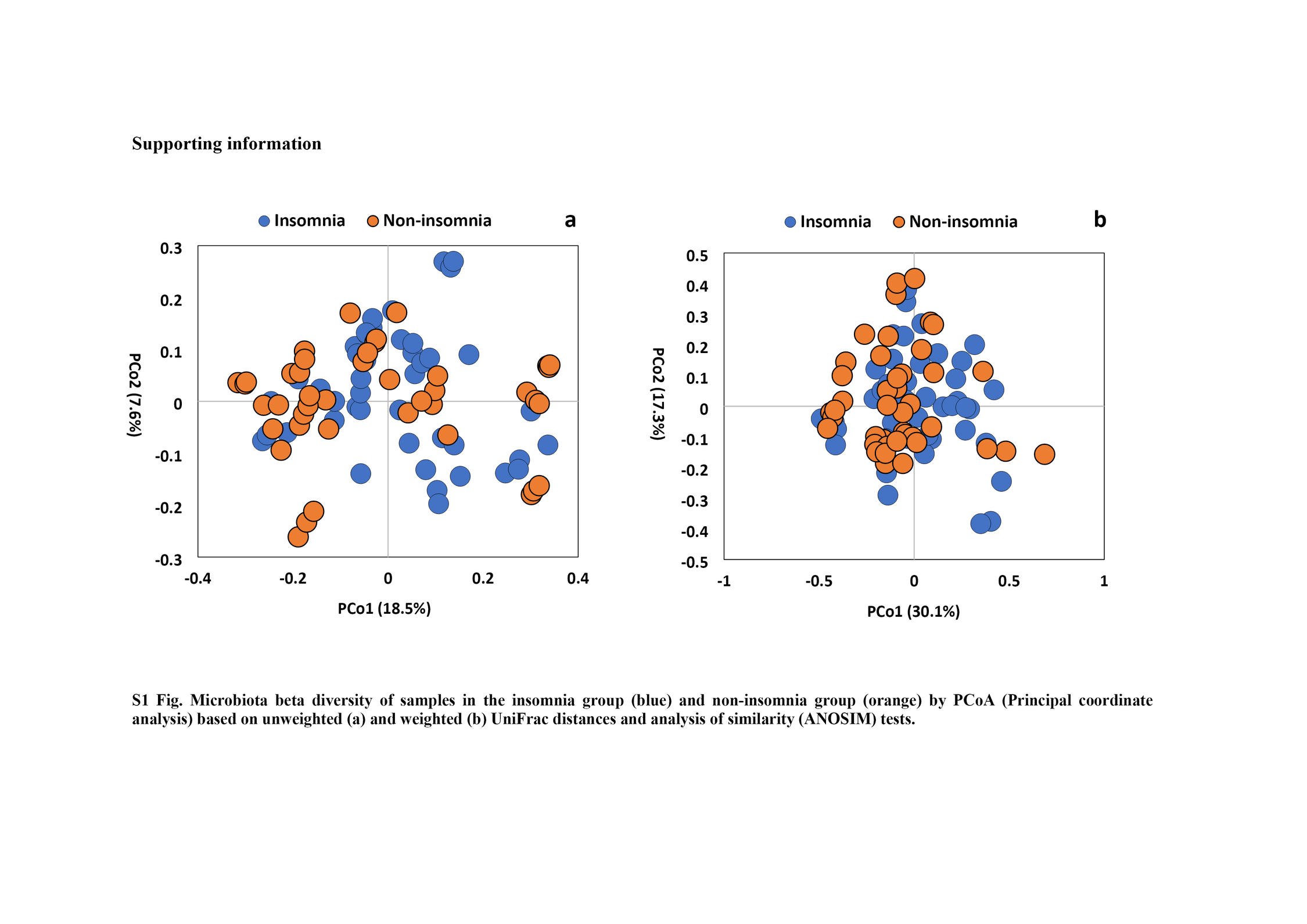

Supplement: S1 Fig — Microbiota beta diversity of samples in the insomnia group (blue) and non-insomnia group (orange) by PCoA (Principal coordinate analysis) based on unweighted (a) and weighted (b) UniFrac distances and analysis of similarity (ANOSIM) tests. (TIF) [file pone.0296047.s001.tif]
